# Increased psychological distress after the lifting of COVID-19 lockdown in the Saudi population: a cross-sectional study

**DOI:** 10.1186/s43045-021-00167-9

**Published:** 2022-01-03

**Authors:** Hatim Yousef Alharbi, Sami S. Alharthi, Ahmed S. Alzahrani, Mohammed Khalid A. Dakhel, Ziyad Hussain Alawaji

**Affiliations:** 1 Department of Psychiatry, College of Medicine, Qassim University, Buraydah, Saudi Arabia; 2 Medicine Department, Taif University, Taif, Saudi Arabia; 3 Preventive Medicine Department, Armed Forced Hospital, Madinah, Saudi Arabia

**Keywords:** COVID-19, Lockdown, Psychological distress, PTSD, Depression, Anxiety, Saudi Arabia

## Abstract

**Background:**

Amid the ongoing COVID-19 pandemic and its global health and socioeconomic aftereffects, the enduring state of crisis is increasingly impacting the coping capacity of the populations. In this study, we aimed to characterize the levels of psychological distress after the lifting of COVID-19 lockdown.

**Results:**

The Impact of Event Scale (IES-R) and Depression, Anxiety, and Stress Scales-21 items (DASS-21) were used to screen for post-traumatic stress syndrome (PTSD), depression, anxiety, and stress. The prevalence of PTSD was 41.6% and was associated with severe or extremely severe stress (27.8%), anxiety (31.4%), and depression (39.0%). All disorders were strongly correlated with one another. The risk of developing PTSD was independently associated with residence in high COVID-19 prevalence region (OR = 2.25, *p* = 0.004), poor (OR = 3.98, *p* = 0.002), or moderate (OR = 1.63, *p* = 0.048) self-assessed overall physical health, psychiatric comorbidity (OR = 1.87, *p* = 0.036), number of COVID-19-like symptoms (OR = 1.94, *p* = 0.039), and severe COVID-19 morbidity in the acquaintances (OR = 1.54, *p* = 0.026). Four theories were proposed to explain these high figures, with a discussion of their practical implications.

**Conclusions:**

The lifting of lockdown measures was associated with a substantial increase in psychological distress among the Saudi population, referring to figures reported during the lockdown. This may indicate a decline in the overall population’s coping capacity with the enduring crisis.

**Supplementary Information:**

The online version contains supplementary material available at 10.1186/s43045-021-00167-9.

## Background

Amid the ongoing COVID-19 pandemic and its global health and socioeconomic aftereffects, the enduring state of crisis is increasingly impacting the coping capacity of the populations. Plenty of national and international reports outlined the immediate and delayed psychological adverse effects of both the pandemic and the lockdown and restrictive measures imposed on the individuals and the consequent abrupt change in lifestyle [1–7]. The extent of such impact prompted scientists and visionaries to question the relevance and levels of restrictive measures in the long term and their rebound effect on the environment and economic perspectives [8, 9].

Approximately, 1 year after the pandemic, the rise in COVID-19 deaths and new cases with the emergence and spread of a mutant strain, add up to the worries and skepticism of the populations regarding the efficacy of the vaccination to put an end to the crisis [10–13]. This probably breeds fears among the people and raises worries about another imminent lockdown and all the psychological and socioeconomic burdens it comprises.

A brief flashback on the timeline of the first lockdown measures implemented in the Kingdom of Saudi Arabia in the first months of the pandemic shows 3 phases: (1) start of the curfew on 23 March; (2) phase 1 re-opening on 28 May; and (3) drop of all restrictions—except social distancing and universal masking—on 21 June. Several authors addressed the psychological impact of the restrictive measures on the Saudi population during the period 23 March—21 June 2020 [14–16]. However, no study explored the delayed psychological impact of such measures, notably the levels of post-traumatic stress after the restrictions drop or whether the levels of psychological distress decreased. That is, while these restrictive measures have probably enabled flattening the epidemic curve, the cumulative number of COVID-19 cases and deaths may have reached a worrying level for the population. Such figures may induce a paradoxical effect of lockdown lifting. On the other hand, the relative return to normal life may have relieved the anxiety and stress.

We conducted the present study to characterize the levels of psychological distress 1 week after lifting the COVID-19 lockdown in Saudi Arabia. We assessed post-traumatic stress disorder (PTSD), depression, anxiety, and stress levels and analyzed sociodemographic and epidemiological factors. We further studied the inter-correlations between the four disorders.

## Methods

### Design and population

A cross-sectional study was conducted between 28 June and 5 July 2020. It involved adult individuals residing in the Kingdom of Saudi Arabia during the study period. Individuals with age below 18 years and those who are not speaking the Arabic language were not included.

### Tools

The presence and severity of PTSD were estimated using the revised version of the Impact of Event Scale (IES-R), which consists of a 22-item questionnaire assessing the extent of the difficulty experienced after stressful life events in various dimensions, using 5-level Likert-type questions [17]. An Arabic version that was previously developed and validated by the co-author of the present study was used [18]. The IES-R score is calculated, and a cutoff value ≥ 33 was considered to define PTSD diagnosis [19].

Anxiety and depression disorders were diagnosed using the Arabic version of the Depression, Anxiety and Stress Scale-21 (DASS-21), which showed good psychometric proprieties [20, 21]. The scale comprises 21 questions, 7 for depression, 7 for anxiety, and 7 for stress, each using a 4-level Likert-type scale to rate, from 0 to 3, the level of applicability of the given statement to the participant. A final score is calculated, and specific cutoffs enable defining five levels of severity of each disorder, including normal, mild, moderate, severe, and extremely severe [22]. For the present study, severe and extremely severe levels were considered to define positive depression, anxiety, or stress cases.

Besides IES-R and DASS-21, a semi-structured questionnaire was developed to collect the participants’ sociodemographic, health-related, and epidemiological factors. Sociodemographic factors included gender, age category, educational level, marital status, professional status, number of children, family income, and residence location. Health-related factors included the presence of chronic diseases, psychiatric comorbidity prior COVID-19 among a list of 9 disorders (PTSD, anxiety, depression, sleep disorder, etc.), self-rated overall physical health (poor=1 to excellent=5), clinic visit or hospitalization during the last 14 days, and occurrence of evocative symptoms of COVID-19 during the past 14 days among a predefined list of 11 symptoms. Epidemiological data included direct or indirect contact with confirmed or suspect cases, contact with contaminated material, screening for COVID-19 during the past 14 days, quarantine in the past 14 days, and COVID-19-related death or ICU admission in the acquaintances.

### Procedure

The questionnaire was edited for online administration using the Google Form platform. In addition, the link was disseminated through the most frequently used social media such as Facebook, WhatsApp, Twitter, and Instagram.

### Statistical methods

Statistical analysis was performed with the Statistical Package for Social Sciences version 21.0 for Windows (SPSS Inc., Chicago, IL, USA). Categorical variables are presented as frequency and percentage, while continuous variables are presented as mean ± standard deviation (SD). The internal consistency of the IES-R and DASS-21 scales was analyzed by calculating Cronbach’s alpha. The bivariate correlations of IES-R, depression, anxiety, and stress scores with one another were analyzed by calculation of Pearson’s *R* coefficient (*R*). Chi-square test was used to analyze the associations of severity levels of PTSD, depression, anxiety, and stress with one another; results are presented as cross-tabulations with the significance level.

Furthermore, as appropriate, the bivariate associations of PTSD with the different sociodemographic, clinical, and epidemiological factors were analyzed using chi-square and Fisher’s exact tests. Finally, a multivariate binary logistic regression was carried out to determine the independent factors of PTSD among those which showed significant association in bivariate analysis; results are presented as odds ratio (OR) with 95% confidence interval (95% CI). A *p* value of < 0.05 was considered to reject the null hypothesis.

## Results

### Participants’ characteristics

A total of 1323 complete participation were received; however, due to overrepresentation of the 18-30 years age category (84.1%), a post-stratification was conducted to create an age-standardized sample by reference to the general population characteristics. This was carried by randomly selecting 300 participants out of the 1113 from the 18-30 years category, which resulted in a final sample size = 510. Demographic characteristics of the 510 included are depicted in Table 1 and showed the predominance of young (18-30 years, 58.8%), males (59.2%) with high education (University of higher, 68.4%). Distribution by region was discrepant, with higher participation from Riyadh (27.6%), the Holy City of Makkah (23.3%), and Al Qassim (21.2%). Only 12.4% of the participants were working in the health sector.

**Table 1:** Participants’ characteristics (*N* = 510)

Parameter	Category	N	%
Gender	Male	302	59.2
	Female	208	40.8
Age category (years)	**18-30**	300	58.8
	31-40	128	25.1
	41-50	58	11.4
	51-60	20	3.9
	> 60	4	0.8
Educational level	Primary	2	0.4
	Middle	7	1.4
	Secondary	152	29.8
	**University**	282	55.3
	Diploma	40	7.8
	Master	20	3.9
	PhD	7	1.4
Region/province	Makkah	119	23.3
	Madinah	37	7.3
	Riyadh	141	27.6
	Qassim	108	21.2
	Eastern Province	32	6.3
	Northern regions	20	3.9
	Southern regions	29	5.7
	Moving	24	4.7
Marital status	Single	291	57.1
	Married	208	40.8
	Divorced	10	2.0
	Widow	1	.2
Number of children	None	302	59.2
	1-3	115	22.5
	4-6	83	16.3
	7-10	10	2.0
Professional status	Unemployed	71	13.9
	Student	225	44.1
	Employee	188	36.9
	Entrepreneur	14	2.7
	Retired	12	2.4
Working in health sector	No	447	87.6
	Yes	63	12.4
Relative working in health sector	No	351	68.8
	Yes	159	31.2
Family income (SAR)	< 5k	67	13.1
	5-10k	130	25.5
	10-15k	136	26.7
	15-20k	78	15.3
	20-25k	41	8.0
	> 25k	58	11.4
Accommodation	Apartment	198	38.8
	Floor	105	20.6
	Villa	207	40.6
No. occupants	1	9	1.8
	2	16	3.1
	3-5	173	33.9
	6+	312	61.2

### Clinical and epidemiological characteristics

Of the total participants, 31.0% reported having suffered headaches during the COVID-19 lockdown, and 44.3% reported at least one of the symptoms evocative for COVID-19. Other epidemiological data showed 7.6% and 7.8% direct and indirect contact cases with confirmed COVID-19 persons, respectively. Medical history showed chronic disease (13.7%), psychiatric comorbidity (11.4%), and a recent visit to the physician (23.7%). Approximately, 1 out of 10 (10.2%) were screened for COVID-19 in the past 14 days, 7.1% were quarantined, and 41.2% declared having a person who has died or been in ICU from COVID-19 (Table 2). Details of psychiatric comorbidities are presented in Table 3.

**Table 2:** Risk factors and epidemiological data (*N* = 510)

Dimension/factor	***N***	%
***COVID-19-like symptoms*** **during the past 14 days**^a^		
Headache	158	31.0
Myalgia	98	19.2
Nasal congestion	61	12.0
Sore throat	57	11.2
Vertigo	47	9.2
Shortness of breath	40	7.8
Diarrhea	39	7.6
Fever > 1 day	25	4.9
Dry cough	19	3.7
Other digestive complaints	2	0.4
Anosmia-Ageusia	1	0.2
**Number of COVID-19-like symptoms**		
None	284	55.7
1	83	16.3
2	61	12.0
3+	82	16.1
Direct contact with a confirmed case	39	7.6
Indirect contact with a confirmed case	40	7.8
Contact with the suspect case	51	10.0
Contact with contaminated material	13	2.5
Not sure	146	28.6
**Self-reported overall physical health**		
Poor	5	1.0
Below moderate	23	4.5
Moderate	92	18.0
Optimal	144	28.2
Excellent	246	48.2
Chronic disease	70	13.7
Psychiatric comorbidity^b^	58	11.4
Clinic or physician consultation in the past 14 days	121	23.7
Hospitalization in the past 14 days	4	0.8
Screening for COVID-19 in the past 14 days	52	10.2
Quarantined in the past 14 days	36	7.1
COVID-19 related death or ICU admission in the acquaintances	210	41.2

^a^A participant may experience more than one symptom

^b^Psychiatric history is detailed in Table 3

**Table 3:** Psychiatric comorbidity in the study population

Psychiatric comorbidity	*N*	%
PTSD	9	1.8
Anxiety disorder	38	7.5
Depression disorder	40	7.8
Compulsive disorder	16	3.1
Phobia	4	0.8
Eating disorder	17	3.3
Sleep disorder	43	8.4
Personality disorder	4	0.8
Panic disorder	2	0.4
Other	62	12.2

### Assessment of PTSD, depression, anxiety, and stress

All scales showed good reliability in the study population, with Cronbach’s alphas for IES-R (0.923, 22 items), depression scale (0.896), anxiety scale (0.816), and stress scale (0.894). The calculated scores showed mean ± SD for PTSD (IES-R, 28.45 ± 18.00), stress (16.74 ± 12.63), anxiety (10.84 ± 10.21), and depression (16.98 ± 13.16). The prevalence of PTSD (IES-R score ≥ 33) was 41.6% (95% CI=37.3-46.0%). There were high percentages of severe or extremely severe stress (27.8%), anxiety (31.4%), and depression (39.0%), which were associated with significantly higher prevalence (> 63%) of PTSD (Table 4). Further, there was a positive correlation of IES-R score with stress (*R* = 0.674), anxiety (*R* = 0.648), and depression (*R* = 0.608) scores. Stress, anxiety, and depression scores were strongly correlated with each other (*R* = 0.725, 0.782, and 0.831).

**Table 4:** Levels and incidence of post-traumatic stress disorder and anxiety and depression following COVID-19 lockdown (*N* = 510)

Disorder	Severity (score)	N	%	Prevalence^**§**^ of PTSD (IES-R score ≥ 33)		
				***N***	%	***p*** value
**PTSD**	No PTSD (0-23)	215	42.2	-		
	Partial (24-32)	83	16.3			
	Probable (33-36)	41	8.0			
	Severe (37+)	171	33.5			
**Stress**	Normal	262	51.4	37	14.1	
	Mild	34	6.7	16	47.1	
	Moderate	72	14.1	45	60.5	
	Severe	73	14.3	56	76.7	
	Extremely severe	69	13.5	58	84.1	< .001*
**Anxiety**	Normal	241	47.3	38	15.8	
	Mild	37	7.3	11	29.7	
	Moderate	72	14.1	37	51.4	
	Severe	48	9.4	32	66.7	
	Extremely severe	112	22.0	94	83.9	< .001*
**Depression**	Normal	189	37.1	29	15.3	
	Mild	48	9.4	15	31.3	
	Moderate	74	14.5	26	35.1	
	Severe	65	12.7	41	63.1	
	Extremely severe	134	26.3	101	75.4	< .001*

^§^Numbers and percentages are calculated on the corresponding row category

*Statistically significant result (*p* < 0.050)

### Sociodemographic factors associated with PTSD

The prevalence of PTSD was highest in Eastern Province (62.5%), followed by Al Madinah (54.1%), both were knowledge to have a high prevalence risk of COVID-19 (> 10,000 cases per 1 million inhabitants). By classifying the regions into three categories, we observed a significant association of the prevalence of PTSD with the prevalence risk of COVID-19 in the region (*p* = 0.020). No association of PTSD prevalence was found with gender, age, educational level, profession, or family income (Table 5). Comparable associations were found with the prevalence of severe or extremely severe depression, anxiety, and stress as determined using DASS-21 (Table 1S).

**Table 5:** Sociodemographic factors associated with PTSD post COVID-19 lockdown

Parameter	Category	N	%	*p* value
Gender	Male	128	42.4	
	Female	84	40.4	.653
Age category (years)	18-30	123	41.0	
	31-40	57	44.5	
	41-50	27	46.6	
	> 50	5	20.8	.148
Educational level	Primary	1	50.0	
	Middle	2	28.6	
	Secondary	64	42.1	
	University	116	41.1	
	Post-graduate	29	43.3	.955
Region/province	Makkah	48	40.3	
	Madinah	20	54.1	
	Riyadh	51	36.2	
	Qassim	46	42.6	
	Eastern Province	20	62.5	
	Northern regions	4	20.0	
	Southern regions	11	37.9	
	Moving	12	50.0	.041*
Region prevalence (cases per 1 million inhabitants)	Low (< 10k)	59	34.9	
	Moderate (10-12k)	95	40.8	
	High (> 12k)	46	54.8	
	Unclassified (moving)	12	50.0	.020*
Marital status	Single	123	42.3	
	Married	84	40.4	
	Divorced	5	50.0	
	Widow	0	0.0	.757
Number of children	None	126	41.7	
	1-3	51	44.3	
	4-6	31	37.3	
	7-10	4	40.0	.804
Professional status	Unemployed	29	40.8	
	Student	93	41.3	
	Employee	82	43.6	
	Entrepreneur	4	28.6	
	Retired	4	33.3	.799
Working in health sector	No	187	41.8	
	Yes	25	39.7	.746
Relative working in health sector	No	154	43.9	
	Yes	58	36.5	.116
Family income (SAR)	< 5k	35	52.2	
	5-10k	55	42.3	
	10-15k	56	41.2	
	15-20k	24	30.8	
	20-25k	21	51.2	
	> 25k	21	36.2	.102
Accommodation	Apartment	87	43.9	
	Floor	46	43.8	
	Villa	79	38.2	.435
No. occupants	1	3	33.3	
	2	5	31.3	
	3-5	77	44.5	
	6+	127	40.7	.645

*Statistically significant result (*p* < 0.050)

### Epidemiological and health-related factors associated with PTSD

Of the 11 explored symptoms, 6 were likely to be associated with higher prevalence of PTSD; these included fever (60.0% vs 40.6%, *p* = 0.055), sore throat (52.6% vs 40.2%, *p* = 0.072), difficulty breathing (55.0% vs 40.4%, *p* = 0.073), headache (51.3% vs 37.2%, *p* = 0.003), myalgia (54.1% vs 38.6%, *p* = 0.005), and dry cough (57.9% vs 40.9%, *p* = 0.141), versus absence of the symptom, respectively. These symptoms were designated as “alarming symptoms,” and their number was significantly associated with a higher percentage of PTSD (*p* = 0.019). Likewise, the percentage of PTSD was associated with the presence of chronic disease (54.3% vs 39.5%, *p* = 0.020) or psychiatric comorbidity (56.9% vs 39.6%, *p* = 0.012), versus absence respectively, while it was inversely associated with the self-reported overall physical health status (*p* = 0.001). Notably, PTSD was more frequent among participants who declared having in their acquaintance a person who was dead from or admitted in ICU for COVID-19 (49.0% vs 36.3%, *p* = 0.004), compared with those who had none respectively (Table 6). Comparable associations were found with the prevalence of severe or extremely severe depression, anxiety, and stress as determined using DASS-21 (Table 2S).

**Table 6:** Health-status and epidemiological factors associated with PTSD post-COVID-19 lockdown (*N* = 510)

Factor	Level	***N***	%	***p*** value
No. of alarming symptoms	None	113	36.6	
	1	42	45.2	
	2	28	49.1	
	3+	29	56.9	.019*
Direct contact with a confirmed case	No	195	41.4	
	Yes	17	43.6	.790
Indirect contact with a confirmed case	No	199	42.3	
	Yes	13	32.5	.225
Contact with the suspect case	No	190	41.4	
	Yes	22	43.1	.811
Contact with contaminated material	No	205	41.2	
	Yes	7	53.8	.363
Not sure	No	94	42.5	
	Yes	118	40.8	.699
Self-reported overall physical health	Low	20	71.4	
	Moderate	48	52.2	
	Optimal or excellent	144	36.9	< .001*
Chronic disease	No	174	39.5	
	Yes	38	54.3	.020*
Psychiatric comorbidity	No	179	39.6	
	Yes	33	56.9	.012*
Clinic or physician consultation in the past 14 days	No	161	41.4	
	Yes	51	42.1	.882
Hospitalization in the past 14 days	No	210	41.5	
	Yes	2	50.0	.731^F^
Screening for COVID-19 in the past 14 days	No	193	42.1	
	Yes	19	36.5	.437
Quarantined in the past 14 days	No	197	41.6	
	Yes	15	41.7	.990
Person dead from or admitted in ICU for COVID-19 in acquaintances	No	109	36.3	
	Yes	103	49.0	.004*

*Statistically significant result (*p* < 0.050); test used

^F^Fisher’s exact test, otherwise, chi-square test was used

### Predictors of PTSD

The risk of developing PTSD was independently associated with residence in high COVID-19 prevalence region (OR = 2.25, *p* = 0.004), poor (OR = 3.98, *p* = 0.002) or moderate (OR = 1.63, *p* = 0.048) self-assessed overall physical health, psychiatric comorbidity (OR = 1.87, *p* = 0.036), having developed more than three alarming symptoms (OR = 1.94, *p* = 0.039), and having in the acquaintance a person who was dead from or admitted in ICU for COVID-19 (OR = 1.54, *p* = 0.026) (Table 7).

**Table 7:** Independent factors associated with PTSD post COVID-19 lockdown

Factor	Category	OR	95% CI		***p*** value
COVID-19 prevalence in location	Low	Ref	-	-	.021*
	Moderate	1.25	0.81	1.92	.310
	High	2.25	1.29	3.93	.004*
	Unclassified	2.23	0.92	5.41	.077
Self-assessed overall physical health	Poor	3.98	1.66	9.54	.002*
	Moderate	1.63	1.00	2.64	.048*
	Optimal or excellent	Ref	-	-	.002*
Chronic disease	Yes	1.56	0.92	2.67	.102
Psychiatric history	Yes	1.87	1.04	3.34	.036*
Person dead or admitted in ICU for COVID-19 in acquaintances	Yes	1.54	1.05	2.24	.026*
Alarming symptoms	Nil	Ref	-	-	.086
	1	1.40	.85	2.31	.181
	2	1.65	.91	2.98	.096
	3+	1.94	1.03	3.64	.039*

Multivariate logistic regression: dependent variable = post-traumatic stress syndrome

*OR* odds-ratio, *95% CI* 95% confidence interval, *Ref* category used as reference in the regression equation

*Statistically significant result (*p* < 0.050)

## Discussion

### Prelude

As the world is facing a recent exacerbation of new COVID-19 cases and deaths, the shadow of a new lockdown looms over the populations, carrying the fears and worries of a dark future for many people [22–24]. Yet, the psychological sequelae of the first lockdown are not completely healed since the return to the normal life is long in coming while the damage is severe. The major findings of the present study provide strong indications regarding the psychological impact of the COVID-19 crisis and the associated restrictive lockdown measures in a society where psychiatric and psychological care is not common and where the religious and social conventions endorse resilience and reliance on God as the main coping strategies, both with everyday life stressors and sudden changes in well-being [25–27].

### Increased psychological distress after the lifting of COVID-19 lockdown in the Saudi population

Both IES-R and DASS scales performed well in the study population. These scales showed a high prevalence of PTSD 1 week after lifting the first COVID-19 lockdown, which was significantly associated with high levels of stress, anxiety, and depression disorders. On the other hand, there was no disparity of PTSD or stress, anxiety, and depression disorders across the different sociodemographic factors. The major observation is that PTSD and depression figures found in the present study were higher than those found during the lockdown period. A study by Alshehri et al. found a prevalence of PTSD of approximately 25% using the PTSD checklist (PCL-5) for Diagnostic and Statistical Manual of Mental Disorders 5 (DSM-5) criteria. Additionally, the study showed significant associations of the prevalence of PTSD with sociodemographic factors as it was higher in females, single participants, and low-income classes [15]. Likewise, Alkhamees et al. observed lower rates of PTSD (23.6%), and severe or extremely severe stress (13.7%), anxiety (13.9%), and depression (17.4%) using IES-R and DASS-21. Furthermore, Alkhamees et al. observed significantly higher scores among females and younger age categories in all disorders [16]. Another study by Alghamdi et al. used DASS-21 to assess psychological distress during the 5th week of the complete curfew among the public, healthcare workers, and security force personnel in Saudi Arabia. Authors found comparable prevalence rates of severe or extremely severe depression (13% to 17%) and anxiety (22.5% to 25%) between the three subpopulations, whereas security force personnel had relatively lower levels of stress (~7%) compared to the two other categories (14.5%-18%) respectively [14]. Overall, the levels of PTSD and depression reported in the present study are higher than those reported in other Saudi studies conducted during the curfew period. Several factors may contribute to the rise of psychological distress indices found in the present study. We propose four main theories that may competitively explain these figures. These theories are the following:

1. Overestimation due to inappropriateness of the scales
2. The hugeness of the epidemiological picture and or severity of the restrictive measures
3. The snowballing proportion of vulnerable individuals
4. Increase in risk perception among the population after the lifting of the curfew and the recognition of the extent of the pandemic

The following sections discuss these theories and provide directions toward their practical implications in light of the literature and the present study findings.

### Overestimation: Are the used PTSD scales appropriate in COVID-19?

By focusing on PTSD, the first theory to explain the high levels consists of questioning the validity of the assessment tools and their appropriateness in the context of COVID-19. Internationally, the prevalence of clinically significant PTSD during the COVID-19 crisis was variable and did not seem to be consistent with the epidemiological figures. For example, in Italy, where the crisis was remarkably severe, a national online study, using the PCL-5 scale, found 20% of cases with significant PTSD symptoms among 1321 participants, which was positively associated with anxiety and depression symptoms. In contrast to our study, the Italian study found a statistically significant association of PTSD with gender, educational level, and positive COVID-19 contacts [28]. On the other hand, significantly higher figures were reported from other countries. A Lebanese study that used the PTSD Checklist–Civilian Version (PCL-C) to screen for PTSD symptoms among Lebanese citizens found a very high prevalence of symptoms, 2 weeks after the start date of the lockdown, notably numbing symptoms characterized by avoidance and passivity (up to 43.4%) and active symptoms (up to 33.2%). The same study showed a remarkable increase in the prevalence of symptoms in the 4th week of lockdown, exceeding 60% [29]. In Portugal, a recent study showed similarly high rates of severe PTSD (42.3%), which is comparable to findings of our study; however, significantly lower rates of severe or extremely severe depression (1.1%), anxiety (6.2%), and stress (0.0) were reported [30]. Similar to our study, the Portuguese study used IES-R and DASS-21 scales.

The PTSD figures found in the present study and other studies may be overestimated due to a potential inappropriateness of the screening tools to the case of an ongoing crisis. By looking into the 22 IES-R items, at least 10 of them may be misinterpreted and result in false-positive responses. For example, a positive answer to items 1 (any reminder brought back feelings about it), 5 (I avoided letting myself get upset when I thought about it or was reminded of it), or 21 (I felt watchful and on-guard) may be confounded with the effect of the continuous flow of breaking news on the pandemic, an actual socioeconomic impact such as income decrease, or the fear of being infected, respectively. It is interesting to note that an Italian team developed a COVID-19-specific tool to screen for PTSD, which found a prevalence of PTSD symptomatology as high as 27.5% that correlated with other indicators of psychological health, including general distress (*r* = 0.77) and sleep disturbance (*r* = 0.53). On the other hand, the Italian COVID-19-PTSD scale correlated well with the IES-R overall scale (*r* = 0.70) and subscales (*r* = 0.39 to 0.66) [31]. Furthermore, the risk mentioned above of overestimation and false positivity should be considered, especially in online-based studies. It would be of considerable interest to study the sensitivity and specificity of the scales used to assess psychological distress during COVID-19 by reference to clinical diagnosis by a psychiatrist.

### The hugeness of the epidemiological picture or severity of the restrictive measure?

Remarkably, the highest rates of PTSD were observed in the Eastern Province and Al Madinah, both having been subject to stricter lockdown measures inducing more prolonged and more stringent movement restrictions due to the higher number of COVID-19 cases in the first mass screening data [32]. By contrast, the lowest prevalence of PTSD was found in regions with the lowest prevalence of COVID-19, notably the southern regions. Another national study confirmed this, which showed a significantly higher prevalence of PTSD in the Eastern province (32.2%), while the lowest rates were reported in the Southern region (18.7%) [15]. This difference between the regions may be explained by the severity of lockdown and curfew measures. According to community mobility reports, the most substantial decline in the population mobility in the Eastern Province and Al Madinah was in April-May 2020 and reached down to −91% from baseline [33]. This suggestion is supported by a study from the USA, which showed higher levels of psychological distress in populations from states that implemented more restrictive lockdown and curfew policies [34]. This constitutes a critical public health indicator of interest for policymakers, health care providers, and individuals, directing the need for preventive actions and psychological support solutions to be implemented in the regions with the highest risk of stricter lockdown.

To this day, nearly 1 year after the start of the pandemic, both the Eastern and Al Madinah Provinces show the highest cumulative prevalence rates, with more than 18k and 14k cases per 1 million people, respectively [35]. Updated data (31 January 2021) from community mobility reports per region show a −29% and −32% decline in park visits in the two provinces versus a national average of −23% [36]. In the meantime, other regions are witnessing an even more substantial decline in mobility, such as Al Jowf, Hail, and Jazan, despite their relatively lower prevalence rates [35, 36]. Such persistence of reduced mobility despite lifting the restrictions may indicate an overall shift in the lifestyle. Yet, the possibility of this being due to the crisis’ socioeconomic and psychological adverse effects or denoting an alienation to social distancing should be raised. Alienation is defined as the loss of personal and social connections in the context of recurrent negative emotions, leading to feelings of powerlessness, meaninglessness, normlessness, isolation, and self-estrangement [17, 37]. There is a strong association of alienation with the occurrence of PTSD symptoms, and this was notably observed in response to the social distancing during the COVID-19 [38]. Furthermore, it was suggested that persistent stress and PTSD are part of a vicious cycle inducing immunosuppression, which may increase susceptibility to COVID-19 infection [39]. Consequently, monitoring the population’s psychological and social well-being is highly recommended, especially in regions subject to more stringent mobility-restricting measures.

### The snowballing proportion of vulnerable individuals

In the present study, psychiatric comorbidity was reported by 11.4% of the participants and was independently associated with an 87% increase in the risk of PTSD. In their study on the Saudi population, Alshehri et al. found that having a psychiatric condition was independently associated with an even higher risk (OR > 3) of developing PTSD during the COVID-19 pandemic, as demonstrated in their stepwise multivariate regression [15]. Comparably, Alkhamees et al. evidenced a positive correlation between a positive psychiatric history and IES-R, DASS-stress, anxiety, and depression scores [16]. Several authors have probed into the hypothesis of whether individuals with psychological and psychiatric disorders are experiencing more psychological distress during the COVID-19 crisis and lockdown. A case-control study that used the IES-R and DASS-21 scales, like our study, showed a higher prevalence of PTSD (31.6% vs 13.8%) and severe or extremely severe anxiety (14.4%% vs 0.9%), depression (13.2% vs 0.9%), and stress (7.8% vs 0.0%) among individuals with psychiatric history compared with their counterparts, respectively. Additionally, the study found a higher prevalence of moderately severe (19.7% vs 1.8%) and severe (7.9% vs 0.9%) clinical insomnia using the insomnia severity index (ISI) [40]. Another case-control study compared individuals with a previous history of depression or suicidal attempts versus those without in terms of the development of depressive disorders, distress, and change in suicidal thoughts during the COVID-19 crisis. Findings showed higher levels of distress and depressive symptoms among both participants with a history of depression (16.5% and 23.3% vs 8.7% and 9.0%) and those with a history of suicidal attempts (16.9% and 38.7% vs 1.9% and 12.0%), compared to their respective controls. Additionally, a significant increase in suicidal thoughts was observed among controls, although lesser than in cases [41]. This denotes the higher vulnerability of psychiatric patients to the COVID-19 crisis and lockdown, which has a significant clinical implication in the management of psychiatric patients. Nevertheless, these observations primarily raise another important public health concern: the rising incidence of psychiatric disorders among the healthy subpopulation, thus snowballing the proportion of vulnerable individuals toward the persisting effects of an enduring crisis. Pressing actions should be undertaken by the health authorities and policymakers to assess the levels of vulnerability and resilience among the subpopulations with low coping capacity and implement preventive strategies against psychological distress among the whole population. As the case may be, with regards to the persistence of the COVID-19 crisis, drastic rearrangements of daily life could be planned to mediate the transformation of coping capacity into adaptive capacity. Such strategies could inspire environmental studies that explored the factors that may enhance the adaptive capacity to climate change among vulnerable and most exposed subpopulations [42].

### Increased risk perception

Findings from the present study suggest that the perceived risk of being infected with COVID-19 could be a major factor of psychological distress. This is demonstrated via three parameters, including the number of alarming symptoms experienced by the participant, COVID-19-related death or ICU admission in the acquaintances, and being tested for COVID-19; besides the previously discussed prevalence of COVID-19 in the residential locality.

There was a higher risk of PTSD among participants who experienced COVID-19-like symptoms, notably +94% odd risk among those who reported three or more out of the six symptoms designated as highly alarming for the population. The association between the occurrence of symptoms that may evocate COVID-19 and the psychological distress was demonstrated in other populations; such as in Portugal, where the number of flu-like symptoms experienced in the last 14 days was associated with a substantial increase in the odd risk of developing depression (OR = 1.90), anxiety (OR = 2.71), and stress (OR = 2.69) [30].

Similarly, having a COVID-19-related death or ICU admission in the acquaintances was independently associated with a 54% increase in the odd risk of PTSD. On the other hand, direct or indirect contact with a confirmed or suspect COVID-19 case showed no effect on developing PTSD. This dimension may be equally related to grief due to the loss of a close relative or to the perceived risk of infection. In line with our findings, Alshehri et al. demonstrated a higher prevalence of PTSD among participants who had a family member die due to COVID-19 (OR ~2) and those who were either confirmed or suspected to have been infected. Remarkably, levels of PTSD were higher among participants who were suspected than those who were confirmed to have been infected [15]. The latter observation supports the hypothesis that risk perception and fear of COVID-19 may constitute the major factor of psychological distress during the pandemic, and, on the other hand, a confirmed infected status may help acquire better resilience and adaptation [43, 44].

This is supported by the third parameter, including whether the participant has undergone COVID-19 screening in the past 14 days. Although not statistically significant, the prevalence of PTSD was lower among participants who declared having been tested for COVID-19 in the past 14 days (36.5%) compared to those who were not tested (42.1%). A similar finding was observed in a German study, which showed a likelihood of a decrease in COVID-19-related anxiety and fear of COVID-19 consequences on owns life among individuals who have been tested [43]. Contrariwise, another study from the UK demonstrated a higher propensity to be tested among individuals with a psychiatric history, probably related to higher levels of anxiety leading to more frequent voluntary testing than those without a psychiatric history. However, by excluding participants with a psychiatric history, the incidence rates of self-harming behaviors were significantly increased among the tested individuals versus non-tested ones (24.1% vs 19.3%, respectively). In contrast, the rates of anxiety and depression were comparable between the two groups [45]. We conclude that anxiety may prompt the willingness for COVID-19 testing, while undergoing the test may reduce the anxiety related to the fear of being infected.

Overall, there seems to be a strong correlation of psychological distress with the perceived risk of COVID-19 infection. In the case of our study, the absence of correlation with objective epidemiological factors, such as direct contact with a confirmed case, may be suggestive of a low level of education regarding the actual risks and preventive measures. This suggests that the perceived risk of infection that generates psychological distress is subjective and, most of the time, based on irrational factors.

## Conclusions

The lifting of lockdown and curfew measures during the COVID-19 crisis was associated with a substantial increase in psychological distress among the Saudi population, notably in PTSD and depressive disorders. The impact was more notable in regions with higher COVID-19 prevalence and more stringent mobility restriction measures; this constitutes a critical public health indicator of interest for policymakers, health care providers, and individuals, urging the implementation of specific measures and supportive solutions in such regions. The other worrying aspect of the pandemic is the decline in the overall population’s coping capacity due to the constantly increasing proportion of vulnerable subgroups with the enduring crisis. Therefore, this is probably a good time to develop visionary plans that would enhance the adaptive capacity of the society to a new daily lifestyle.

## Limitations

The present study is limited by the online and self-administration of the two scales, which may be subject to various biases. The major one is the selection bias, which resulted in an overrepresentation of younger individuals and probably frequent internet and social media users. The second bias is the confounding effect of the crisis persistence with the IES-R items, as many of these assess the reminder of a traumatic event that is supposed to be in the past. Finally, additional confusion may result from the actual socioeconomic impact of the crisis, leading to misinterpretation of some of the items. These issues probably limit the generalizability of the findings.

## Supplementary Information

**Additional file 1: Table 1S**. Sociodemographic factors associated with Depression, Anxiety and Stress post COVID-19 lockdown.

**Additional file 2: Table 2S**. Clinical and epidemiological factors associated with Depression, Anxiety and Stress post-COVID-19 lockdown (N=510).

## Data Availability

Data supporting the findings of this study are available from the corresponding author [AH] on request.

## Abbreviations

IES-R: The Impact of Event Scale-Revised
DASS-21: Depression, Anxiety, and Stress Scales-21 items
PTSD: Post-traumatic stress disorder
OR: Odd ratio
SD: Standard deviation
CI: Confidence interval
ICU: Intensive care unit
PCL-5: PTSD checklist for DSM5
DSM-5: Diagnostic and statistical manual of mental disorders 5
ISI: Insomnia severity index
