# Supplementary material for: Increased psychological distress after the lifting of COVID-19 lockdown in the Saudi population: a cross-sectional study
Source: Middle East Curr Psychiatry. 2022 Jan 3;29(1):1. doi: 10.1186/s43045-021-00167-9 (PMC8721643; doi:10.1186/s43045-021-00167-9)
Supplement: Supplementary file 1 — Additional file 1: Table 1S. Sociodemographic factors associated with Depression, Anxiety and Stress post COVID-19 lockdown. [file 43045_2021_167_MOESM1_ESM.docx]

| **Parameter** | **Category** | **Total** | **Stress** | | **Anxiety** | | **Depression** | |
| --- | --- | --- | --- | --- | --- | --- | --- | --- |
|  |  |  | **N (%)** | **p-value** | **N (%)** | **p-value** | **N (%)** | **p-value** |
| Gender | Male | 302 | 77 (25.5) |  | 98 (32.5) |  | 111 (36.8) |  |
|  | Female | 208 | 65 (31.3) | .154 | 62 (29.8) | .527 | 88 (42.3) | .206 |
| Age category (years) | 18-30 | **300** | 87 (29.0) |  | 94 (31.3) |  | 120 (40.0) |  |
|  | 31-40 | 128 | 31 (24.2) |  | 42 (32.8) |  | 52 (40.6) |  |
|  | 41-50 | 58 | 19 (32.8) |  | 20 (34.5) |  | 22 (37.9) |  |
|  | >50 | 24 | 5 (20.8) | .508 | 4 (16.7) | .424 | 5 (20.8) | .305 |
| Educational level | Primary | 2 | 1 (50.0) |  | 0 (0.0) |  | 1 (50.0) |  |
|  | Middle | 7 | 2 (28.6) |  | 2 (28.6) |  | 2 (28.6) |  |
|  | Secondary | 152 | 41 (27.0) |  | 47 (30.9) |  | 56 (36.8) |  |
|  | University | 282 | 74 (26.2) |  | 88 (31.2) |  | 111 (39.4) |  |
|  | Post-graduate | 67 | 24 (35.8) | .553 | 23 (34.3) | .873 | 29 (43.3) | .870 |
| Region/ Province | Makkah | 119 | 32 (26.9) |  | 34 (28.6) |  | 42 (35.3) |  |
|  | Madinah | 37 | 16 (43.2) |  | 13 (35.1) |  | 18 (48.6) |  |
|  | Riyadh | 141 | 38 (27.0) |  | 45 31.9) |  | 57 (40.4) |  |
|  | Qassim | 108 | 23(21.3) |  | 33 (30.6) |  | 37 (34.3) |  |
|  | Eastern Province | 32 | 14 (43.8) |  | 15 (46.9) |  | 18 (56.3) |  |
|  | Northern regions | 20 | 3 (15.0) |  | 2 (10.0) |  | 3 (15.0) |  |
|  | Southern regions | 29 | 6 (20.7) |  | 8 (27.6) |  | 14 (48.3) |  |
|  | Moving | 24 | 10 (41.7) | .030* | 10 (41.7) | .193 | 10 (41.7) | .067 |
| Region Prevalence (cases per 1 million people) | Low (<10k) | 169 | 40 (23.7) |  | 47 (27.8) |  | 62 (36.7) |  |
|  | Moderate (10-12k) | 233 | 57 (24.5) |  | 69 (29.6) |  | 81 (34.8) |  |
|  | High (>12k) | 84 | 35 (41.7) |  | 34 (40.5) |  | 46 (54.8) |  |
|  | Unclassified (moving) | 24 | 10 (41.7) | .005* | 10 (41.7) | .125 | 10 (41.7) | .012* |
| Marital Status | Single | 291 | 85 (29.2) |  | 91 (31.3) |  | 116 (39.9) |  |
|  | Married | 208 | 54 (26.0) |  | 63 (30.3) |  | 76 (36.5) |  |
|  | Divorced | 10 | 2 (20.0) |  | 5 (50.0) |  | 6 (60.0) |  |
|  | Widow | 1 | 1 (100.0) | .316 | 1 (100.0) | .271 | 1 (100.0) | .257 |
| Number of Children | None | 302 | 88 (29.1) |  | 94 (31.1) |  | 123 (40.7) |  |
|  | 1-3 | 115 | 31 (27.0) |  | 40 (34.8) |  | 46 (40.0) |  |
|  | 4-6 | 83 | 22 (26.5) |  | 24 (28.9) |  | 29 (34.9) |  |
|  | 7-10 | 10 | 1 (10.0) | .585 | 2 (20.0) | .691 | 1 (10.0) | .209 |
| Professional Status | Unemployed | 71 | 22 (31.0) |  | 23 (32.4) |  | 32 (45.1) |  |
|  | Student | 225 | 64 (28.4) |  | 70 (31.1) |  | 86 (38.2) |  |
|  | Employee | 188 | 50 (26.6) |  | 61 (32.4) |  | 74 (39.4) |  |
|  | Entrepreneur | 14 | 3 (21.4) |  | 3 (21.4) |  | 4 (28.6) |  |
|  | Retired | 12 | 3 (25.0) | .929 | 3(25.0) | .908 | 3 (25.0) | .593 |
| Working in Health Sector | No | 447 | 128 (28.6) |  | 142 (31.8) |  | 178 (39.8) |  |
|  | Yes | 63 | 14 (22.2) | .288 | 18 (28.6) | .609 | 21 (33.3) | .232 |
| Relative working in health sector | No | 351 | 101 (28.8) |  | 114 (32.5) |  | 142 (40.5) |  |
|  | Yes | 159 | 41 (25.8) | .485 | 46 (28.9) | .424 | 57 (35.8) | .323 |
| Family income (SAR) | <5k | 67 | 22 (32.8) |  | 24 (35.8) |  | 29 (43.3) |  |
|  | 5-10k | 130 | 33 (25.4) |  | 39 (30.0) |  | 50 (38.5) |  |
|  | 10-15k | 136 | 35 (25.7) |  | 44 (32.4) |  | 54 (39.7) |  |
|  | 15-20k | 78 | 22 (28.2) |  | 21 (26.9) |  | 28 (35.9) |  |
|  | 20-25k | 41 | 15 (36.6) |  | 17 (41.5) |  | 18 (43.9) |  |
|  | >25k | 58 | 15 (25.9) | .669 | 15 (25.9) | .512 | 20 (34.5) | .878 |
| Accommodation | Apartment | 198 | 58 (29.3) |  | 63 (31.8) |  | 74 (37.4) |  |
|  | Floor | 105 | 26 (24.8) |  | 37 (35.2) |  | 45 (42.9) |  |
|  | Villa | 207 | 58 (28.0) | .702 | 60 (29.0) | .523 | 80 (38.6) | .642 |
| No. Occupants | 1 | 9 | 1 (11.1) |  | 1 (11.1) |  | 2 (22.2) |  |
|  | 2 | 16 | 4 (25.0) |  | 5 (31.3) |  | 7 (43.8) |  |
|  | 3-5 | 173 | 53 (30.6) |  | 64 (37.0) |  | 74 (42.8) |  |
|  | 6+ | 312 | 84 (26.9) | .548 | 90 (28.8) | .159 | 116 (37.2) | .442 |

* Statistically significant result (p<0.050).
