# Supplementary material for: Increased psychological distress after the lifting of COVID-19 lockdown in the Saudi population: a cross-sectional study
Source: Middle East Curr Psychiatry. 2022 Jan 3;29(1):1. doi: 10.1186/s43045-021-00167-9 (PMC8721643; doi:10.1186/s43045-021-00167-9)
Supplement: Supplementary file 2 — Additional file 2: Table 2S. Clinical and epidemiological factors associated with Depression, Anxiety and Stress post-COVID-19 lockdown (N=510). [file 43045_2021_167_MOESM2_ESM.docx]

| **Dimension/Factor** | **Level** | **Total** | **Stress** | | **Anxiety** | | **Depression** | |
| --- | --- | --- | --- | --- | --- | --- | --- | --- |
|  |  |  | **N (%)** | **p-value** | **N (%)** | **p-value** | **N (%)** | **p-value** |
| No. of alarming symptoms | None | 309 | 79 (25.6) |  | 81 (26.2) |  | 102 (33.0) |  |
|  | 1 | 93 | 25 (26.9) |  | 34 (36.6) |  | 45 (48.4) |  |
|  | 2 | 57 | 15 (26.3) |  | 23 (40.4) |  | 26 (45.6) |  |
|  | 3+ | 51 | 23 (45.1) | .037* | 22 (43.1) | .015* | 26 (51.0) | .007* |
| Direct contact with confirmed case | No | 471 | 133 (28.2) |  | 148 (31.4) |  | 186 (39.5) |  |
|  | Yes | 39 | 9 (23.1) | .490 | 12 (30.8) | .933 | 13 (33.3) | .449 |
| Indirect contact with confirmed case | No | 470 | 130 (27.7) |  | 153 (32.6) |  | 187 (39.8) |  |
|  | Yes | 40 | 12 (30.0) | .751 | 7 (17.5) | .049* | 12 (30.0) | .223 |
| Contact with suspect case | No | 459 | 120 (26.1) |  | 137 (29.8) |  | 177 (38.6) |  |
|  | Yes | 51 | 22 (43.1) | .010* | 23 (45.1) | .026* | 22 (43.1) | .525 |
| Contact with contaminated material | No | 497 | 137 (27.6) |  | 153 (30.8) |  | 195 (39.2) |  |
|  | Yes | 13 | 5 (38.5) | .387 | 7 (53.8) | .077 | 4 (30.8) | .537 |
| Not sure | No | 364 | 102 (28.0) |  | 108 (29.7) |  | 140 (38.5) |  |
|  | Yes | 146 | 40 (27.4) | .887 | 52 (35.6) | .191 | 59 (40.4) | .683 |
| Self-reported overall physical health | Below moderate | 28 | 17 (60.7) |  | 18 (64.3) |  | 19 (67.9) |  |
|  | Moderate | 92 | 39 (42.4) |  | 44 (47.8) |  | 55 (59.8) |  |
|  | Optimal or Excellent | 390 | 86 (22.1) | <.001* | 98 (25.1) | <.001* | 125 (32.1) | <.001* |
| Chronic disease | No | 440 | 121 (27.5) |  | 132 (30.0) |  | 167 (38.0) |  |
|  | Yes | 70 | 21 (30.0) | .665 | 28 (40.0) | .094 | 32 (45.7) | .216 |
| Psychiatric Comorbidity | No | 452 | 118 (25.7) |  | 130 (28.8) |  | 166 (36.7) |  |
|  | Yes | 58 | 26 (44.8) | .002* | 30 (51.7) | <.001* | 33 (56.9) | .003* |
| Clinic or physician consultation in the past 14 days | No | 389 | 105 (27.0) |  | 117 (30.1) |  | 154 (39.6) |  |
|  | Yes | 121 | 37 (30.6) | .442 | 43 (35.5) | .258 | 45 (37.2) | .637 |
| Hospitalization in the past 14 days | No | 506 | 141 (27.9) |  | 158 (31.2) |  | 197 (38.9) |  |
|  | Yes | 4 | 1 (25.0) | .899 | 2 (50.0) | .420 | 2 (50.0) | .651 |
| Screening for COVID-19 in the past 14 days | No | 458 | 129 (28.2) |  | 143 (31.2) |  | 180 (39.3) |  |
|  | Yes | 52 | 12 (25.0) | .629 | 17 (32.7) | .829 | 19 (36.5) | .699 |
| Quarantined in the past 14 days | No | 474 | 132 (27.8) |  | 148 (31.2) |  | 186 (39.2) |  |
|  | Yes | 36 | 10 (27.8) | .993 | 12 (33.3) | .793 | 13 (36.1) | .711 |
| COVID-19 related death or ICU admission in the acquaintances | No | 300 | 69 (23.0) |  | 82 (27.3) |  | 102 (34.0) |  |
|  | Yes | 210 | 73 (34.8) | .004* | 78 (37.1) | .019* | 97 (46.2) | .005* |

- Statistically significant result (p<0.050); test used: ^F^ Fisher’s exact test; otherwise, chi-square test was used.
